# Understanding the Role of the BAI Subfamily of Adhesion G Protein-Coupled Receptors (GPCRs) in Pathological and Physiological Conditions

**DOI:** 10.3390/genes9120597

**Published:** 2018-11-30

**Authors:** Sun Young Moon, Seong-Ah Shin, Yong-Seok Oh, Hyun Ho Park, Chang Sup Lee

**Affiliations:** 1 College of Pharmacy and Research Institute of Pharmaceutical Sciences, Gyeongsang National University, Jinju 52828, Korea; symoon0414@gnu.ac.kr (S.Y.M.); shinsaya@gnu.ac.kr (S.-A.S.); 2 Department of Brain-Cognitive Sciences, Daegu-Gyeongbuk Institute of Science and Technology (DGIST), Hyeonpung-myeon, Dalseong-gun, Daegu 42988, Korea; ysoh2040@dgist.ac.kr; 3 College of Pharmacy, Chung-Ang University, Seoul 06974, Korea; xrayleox@cau.ac.kr

**Keywords:** brain-specific angiogenesis inhibitor, apoptotic cell clearance, tumorigenesis, engulfment

## Abstract

Brain-specific angiogenesis inhibitors (BAIs) 1, 2, and 3 are members of the adhesion G protein-coupled receptors, subfamily B, which share a conserved seven-transmembrane structure and an N-terminal extracellular domain. In cell- and animal-based studies, these receptors have been shown to play diverse roles under physiological and pathological conditions. BAI1 is an engulfment receptor and performs major functions in apoptotic-cell clearance and interacts (as a pattern recognition receptor) with pathogen components. BAI1 and -3 also participate in myoblast fusion. Furthermore, BAI1–3 have been linked to tumor progression and neurological diseases. In this review, we summarize the current understanding of the functions of BAI1–3 in pathological and physiological conditions and discuss future directions in terms of the importance of BAIs as pharmacological targets in diseases.

## 1. Introduction

Brain-specific angiogenesis inhibitor (*BAI*) *1* was initially identified as a p53-inducible gene, specifically expressed in the brain [1], and *BAI2* and *BAI3* are homologous to *BAI1* [2]. Genes *BAI1*, *BAI2*, and *BAI3* are localized in chromosomal regions 8q24, 1p35, and 6q12, respectively [2]. Although the *BAI1* gene contains a p53-binding site, the regulatory role of p53 is not clear. Some studies indicate that *BAI1* is transcriptionally regulated by p53 [1,2], whereas others have revealed that the level of *BAI1* messenger RNA (mRNA) does not correlate with the p53 status [3,4]. Genes *BAI2* and *BAI3* are not known to contain p53-binding sites. Therefore, further studies are necessary to understand the regulation of *BAI* genes and factors involved in the control over the expression of these genes.

Brain-specific angiogenesis inhibitor-1 is widespread in brain tissues, including the hippocampus, basal ganglia, cerebral cortex, and olfactory bulb [5,6,7,8,9]. In particular, neurons and glia in the brain contain the BAI1 protein in abundance [8,9,10,11]. Nonetheless, more recent studies have shown that BAI1 is expressed in other cell types, such as macrophages, skeletal muscle myoblasts, and peritoneal exudate cells, as well as in organs, such as bone marrow, the spleen, the colon, and testes, indicating that BAI1 is expressed at some point in a variety of tissues [5,12].

Brain-specific angiogenesis inhibitor-2 is restrictively expressed in the brain of neonates and adults, although it is sporadically expressed in multiple cell types in a variety of embryonic tissues, including the brain, heart, skeletal muscle, intestine, and thymus [13]. Because BAI2 is abundantly expressed in various fetal and adult brain tissues, BAI2 may have brain-specific functions [5,12]. [12,13] The localization pattern of mouse *Bai2* (*mBai2*) is similar to that of *mBai1* in the brain [13]. In particular, an anti-*mBai2* mRNA probe detects seven mRNA splice variants, some of which lack a part of thrombospondin type-1 repeats (TSRs) or the third cytoplasmic loop of the seven transmembrane domains, suggesting that each alternative splicing product of *Bai2* can produce several proteins during brain development [13]. Another anatomical study has shown that *Bai2*, like *Bai1* and *Bai3*, is preferentially expressed in the muscle-myenteric nerve layer of a gastrointestinal tract isolated from mice [13,14].

Brain-specific angiogenesis inhibitor-3 is expressed in various postnatal and adult human brain tissues. *BAI3* mRNA expression peaks one day after birth [15]. During embryonic stages, the expression of *BAI3* mRNA is limited to the central nervous system, e.g., cerebellar Purkinje cells and hippocampal neurons, unlike other BAI subfamilies [15,16,17,18]. The abundance of BAI3 in the postsynaptic neuron part is related to its involvement in synapse development and/or function [5,11]. In addition to its expression in the brain, *BAI3* mRNA is expressed in various adult human tissues: the heart, testis, and small intestinal tissues [12].

**Figure 1:**
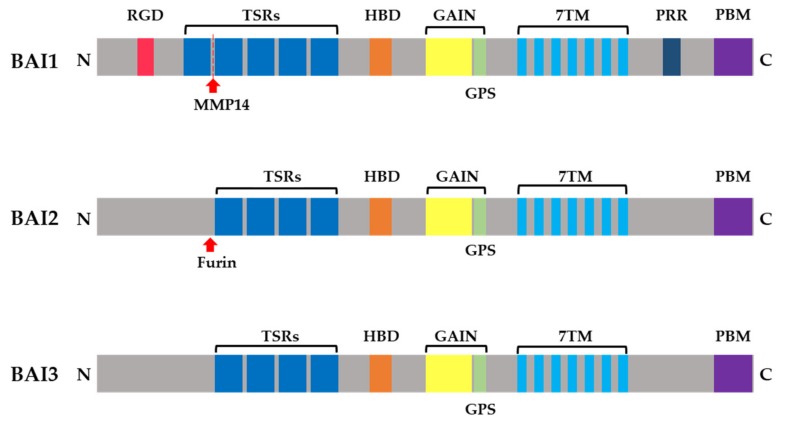
A schematic diagram of protein primary structures of the brain-specific angiogenesis inhibitor (BAI) subfamily (various functional domains). RGD, Arg-Gly-Asp integrin-binding motif; TSR, thrombospondin type 1 repeat; HBD, hormone-binding domain; GAIN, GPCR autoproteolysis-inducing domain; GPS, GPCR proteolytic site; 7TM, seven-transmembrane regions; PRR, proline-rich region; PBM, PDZ-binding motif.

Brain-specific angiogenesis inhibitor-1, -2, and -3 (Figure 1) are members of the adhesion G protein-coupled receptor (GPCR), subfamily B, which share a conserved seven-transmembrane structure and an N-terminal extracellular domain (ECD) and are known to regulate several cellular functions under normal or disease-related conditions [6]. The adhesion GPCRs have a large N-terminal ECD with extracellular binding partners, which may differentially activate receptor signaling and/or mediate cell adhesion [19,20,21]. Many studies have shown that BAI1–3 function through the interaction of their N-terminal region with several specific ligands. Thrombospondin type-1 repeats are dominant in the N termini of all three BAIs. Thrombospondin type-1 repeats are known to be functionally associated with the inhibition of angiogenesis, cell migration, and cell-to-cell attachment [22,23,24,25]. Brain-specific angiogenesis inhibitor-1 contains five TSRs, which can be cleaved by furin-activated matrix metalloproteinase 14 (MMP14) to release Vstat40 (vasculostatin 40), which inhibits angiogenesis [26]. Some studies have also revealed that TSRs are involved in the association of BAI1 with CD36, α_v_β_5_ integrin, and phosphatidylserine (PtdSer) for the inhibition of angiogenesis, inhibition of the proliferation of endothelial cells, and engulfment of apoptotic cells, respectively [10,27,28]. In addition, BAI1 serves as a pattern recognition receptor by interacting with the lipopolysaccharides of gram-negative bacteria to internalize the attached bacteria [29]. Brain-specific angiogenesis inhibitor-2 and -3 contain four TSRs each. For BAI2, the cleavage upstream of its TSRs by furin has been found to activate the nuclear factor of activated T cells (NFAT) pathway [30]. Brain-specific angiogenesis inhibitor-3 is known to regulate the synaptic density through binding of its TSR with the C1ql protein [31]. A putative hormone-binding domain and a GPCR proteolysis site (GPS) are also present in all three BAI ECDs [12]. The hormone-binding domain in the BAI subfamily has not been studied; however, proteolysis of the BAI1 protein at the GPS has been reported. Vasculostatin 120 (Vstat120) is a product of cleavage at a conserved GPS of BAI1 that inhibits the migration of endothelial cells and reduces in vivo angiogenesis [27]. As mentioned above, BAI2 is processed via proteolysis by furin, although the cleavage site is not a conserved GPS [30]. The extracellular proteolytic event for BAI3 is not known at present.

Besides, BAI1–3 contain a PDZ-binding motif QTEV (Gln-Thr-Glu-Val), which interacts with PDZ domain-containing proteins in the C-terminal intracellular portion for intracellular signal transduction [6,12]. Several proteins are known to function via interaction with the PDZ-binding motif in the C terminus of BAI1. MAGI-3 was shown to potentiate extracellular signal-regulated kinases (ERK) signaling in the postsynaptic density [32] and the Par3-Tiam1 complex is known to promote synapse development by controlling Rac1 activation via interaction with the PDZ-binding motif of BAI1 [11]. As mentioned earlier, BAI1 is also known as the engulfment receptor and recognizes PtdSer on apoptotic cells. The C-terminal α-helix region forms a trimeric complex with ELMO and Dock180 and serves for intracellular signaling to promote the engulfment of apoptotic cells [10]. The cytoplasmic domain of BAI2 can associate with GA-binding protein gamma (GABP-γ) and is involved in the transcriptional regulation of VEGF [33]. The C-terminal α-helical region of BAI3 is known to bind to ELMO, resulting in the regulation of dendrite morphogenesis via the activation of small GTPase Rac1 [16].

Although numerous studies have been conducted since BAI1 was first identified, a lot still needs to be elucidated. The molecular roles of BAIs in various pathological and physiological conditions and the molecules specifically interacting with BAIs for intracellular signaling need to be characterized. Here, we review the current understanding of the functions of BAI1, -2, and -3 in pathological and physiological conditions and discuss the importance of BAIs as pharmacological targets for disease treatment.

## 2. Brain-Specific Angiogenesis Inhibitors as Engulfment Receptors and Pattern-Recognition Receptors

### 2.1. Brain-Specific Angiogenesis Inhibitor1 Functions in Professional Phagocytes

**Figure 2:**
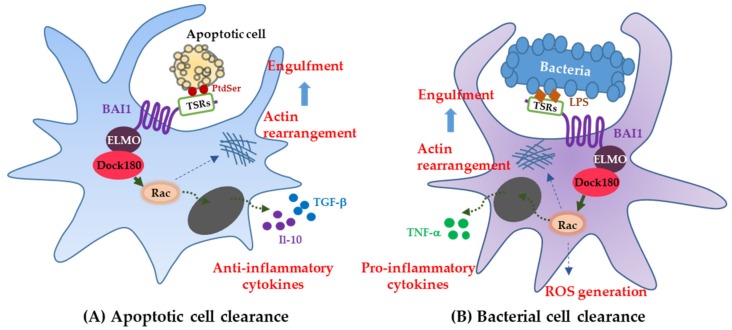

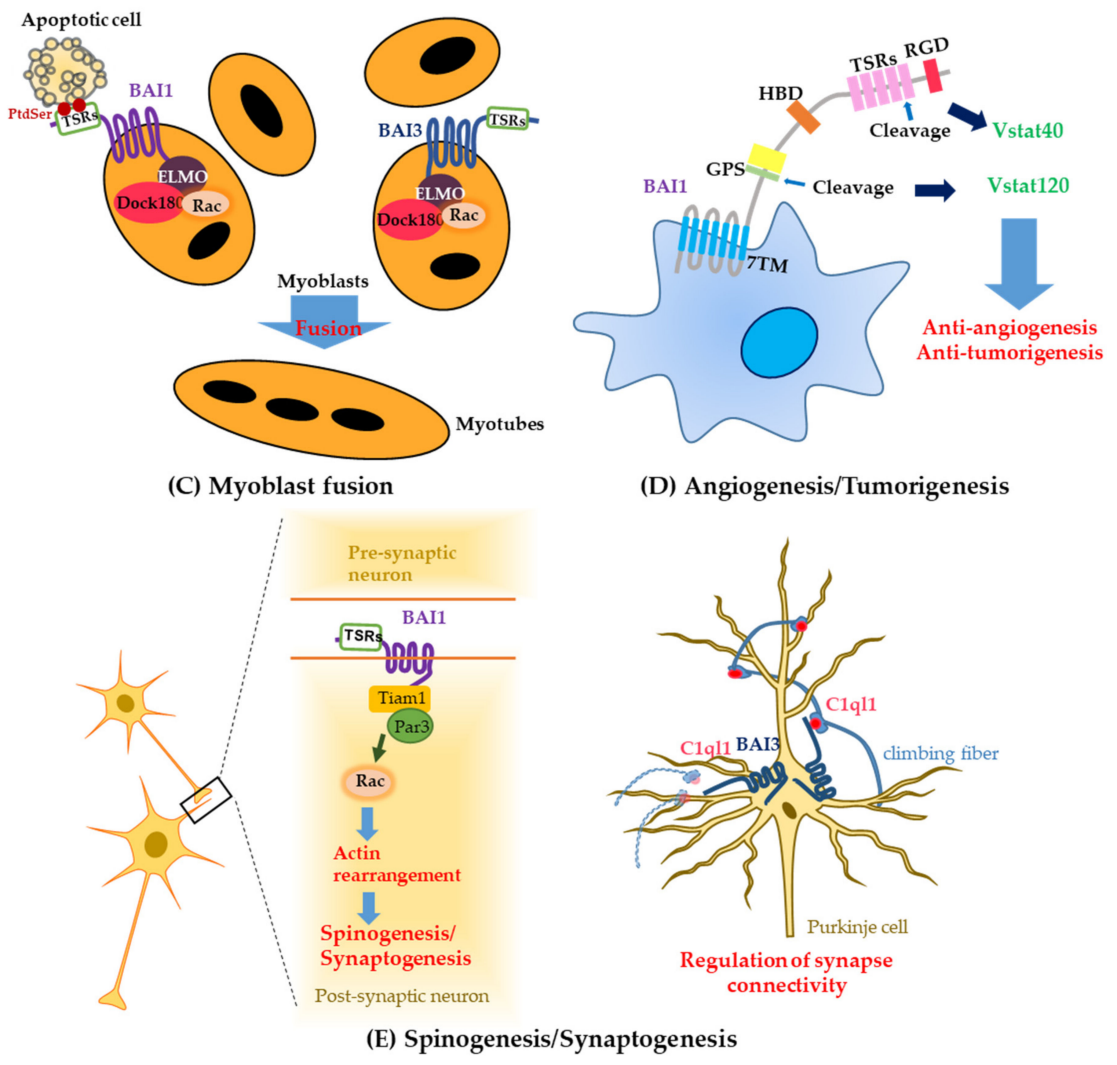
Functions of the BAI subfamily. (**A**) BAI1 as an engulfment receptor; (**B**) BAI1 as a pattern recognition receptor; (**C**) BAI1 and -3 as the mediators of myoblast fusion; (**D**) BAI1 as an inhibitor of angiogenesis and tumorigenesis; (**E**) BAI1 and -3 as modulators of synaptogenesis and spinogenesis.

Brain-specific angiogenesis inhibitor-1 was identified as an engulfment receptor that linked to ELMO, Dock180, and Rac modules, for promoting the internalization of apoptotic cell corpses [10]. Originally, BAI1 was believed to only be involved in the inhibition of neovascularization in experimental angiogenesis through its TSR region [1]. Thus, BAI1 was known as one of the orphan receptors whose ligand was not identified. During death processes of normal cells, apoptotic cells undergo surface changes, like the exposure of PtdSer on the outer leaflet of their plasma membrane [34]. Phagocytic cells recognize the exposed PtdSer on apoptotic cells and then engulf them [35]. Proteins ELMO, Dock180, and Rac, as the downstream modules of BAI1, are known to regulate the actin cytoskeleton of phagocytes during engulfment [36,37]. The direct interaction between TSRs of the BAI1 N terminus and exposed PtdSer on apoptotic cells is known to activate the intracellular Rac signaling pathway, which promotes the engulfment of apoptotic cells (Figure 2A). Therefore, the function of BAI1 as a PtdSer receptor has been demonstrated to be a key player in the apoptotic-cell clearance by phagocytes.

Recently, the BAI1-ELMO1-Rac1 signaling pathway was found to contribute to the upregulation of ATP-binding cassette transporter 1 (ABCA1), a known regulatory protein for cholesterol efflux in phagocytes [38]. ABCA1 is upregulated by the engulfment of apoptotic cells by a phagocyte; this upregulation is independent of the sterol-sensing machinery of the liver X receptor (LXR), which is known to be crucial for ABCA1 upregulation [38]. The deficiency of phagocytic receptor BAI1 in *Bai1* KO mice (mice lacking BAI1) reduces ABCA1 induction in macrophages and increases the numbers of apoptotic cells in their aortic roots, and this change correlates with altered lipid profiles. Conversely, ABCA1 upregulation has been detected in the peritoneal macrophages from *Bai1*-overexpressing transgenic mice in response to apoptotic cells. ABCA1 is known to be a critical cholesterol transporter that is essential for high-density lipoprotein (HDL) biogenesis, and higher HDL levels strongly correlate with a lower incidence of cardiovascular disease [39,40,41,42]. This research indicates that the BAI1-ELMO1-Rac1 pathway may be a therapeutic target for raising HDL levels to combat cardiovascular disease. Therefore, apart from its role as an engulfment receptor in the brain, BAI1 functions as an antiangiogenic factor. The expression of BAI1 is decreased by the inhibition of leucine-rich repeat kinase 2 (LRRK2i) in BV-2 mouse microglial cells treated with the human immunodeficiency virus-1 transactivator of transcription (Tat) protein, and the Tat-induced microglial phagocytosis is suppressed by LRRK2 inhibition [43]. Furthermore, BAI1 has been found to actively participate in the neuronal engulfment by microglia in the brain of embryonic zebrafish and is especially involved in the clearance of dying neurons by promoting phagosome formation in microglia [44].

In addition, a recent study revealed that BAI1 can replace Mertk, as another PtdSer receptor in Sertoli cells and in the retinal pigmented epithelium [45]. Overexpression of BAI1 in *Mertk^−/−^* mice rescues impaired phagocytosis of Sertoli cells in testes. Nonetheless, BAI1 overexpression fails to reverse the defect of retinal pigmented epithelium phagocytosis in the eyes of *Mertk^−/−^* mice [45].

Of note, BAI1 has been reported to be a pattern recognition receptor, interacting with bacterial lipopolysaccharide (LPS) for innate immunity and inflammation in host cells, known as macrophages (Figure 2B) [29]. Bacterial recognition via an interaction between BAI1 TSRs and LPS of gram-negative bacteria results in both bacterial engulfment and proinflammatory responses, in contrast to BAI1 being noninflammatory, often inducing anti-inflammatory molecules during apoptotic cell clearance. Similarly, a recent study revealed that BAI1 promotes phagosomal reactive oxygen species (ROS) production via activation of the Rho family guanosine triphosphate (GTPase) Rac1, thereby enhancing the microbicidal activity of macrophages [46]. Brain-specific angiogenesis inhibitor-1 is necessary not only for the recognition and internalization of bacteria by macrophages, but also for activation of the phagosomal nicotinamide adenine dinucleotide phosphate (NADPH) oxidase complex; a key component of the antimicrobial ROS response [46,47]. The NADPH oxidase complex is especially important in patients with chronic granulomatous disease, who have difficulty forming ROS [46,48]. Brain-specific angiogenesis inhibitor-1-deficient macrophages fail to generate ROS in response to several gram-negative pathogens, as do patients with chronic granulomatous disease.

### 2.2. Brain-Specific Angiogenesis Inhibitor1 Functions in Nonprofessional Phagocytes

Besides the function of BAI1 in professional phagocytes, BAI1 was also reported to mediate apoptotic cell clearance by colonic epithelial cells that are nonprofessional phagocytes in vivo [49]. *Bai1^−/−^* mice show an accumulation of uncleared apoptotic cells and inflammatory cytokines within the colonic epithelium and greater severity of acute colitis in a model of dextran sulfate sodium (DSS)-induced colonic inflammation. Conversely, transgenic overexpression of BAI1 attenuates the DSS-induced colitis in vivo. Gut epithelial cells commonly participate in nutrient uptake and in the barrier function [50,51,52]. Nevertheless, colonic epithelial cells play a major role in the engulfment of their dying neighbors, acting like professional phagocytes, and BAI1 is a key player contributing to the regulation of the severity of inflammation within colonic tissue. These phenomena may be strongly related to inflammatory bowel diseases, such as Crohn’s disease and ulcerative colitis [53,54], and a new approach to augmenting apoptotic-cell clearance may serve as a therapeutic method for patients with inflammatory bowel diseases.

## 3. The Additional Function of Brain-Specific Angiogenesis Inhibitor1 and Brain-Specific Angiogenesis Inhibitor3 in Myoblast Fusion

Brain-specific angiogenesis inhibitor-1 is well-known for its involvement in apoptotic-cell clearance, which is mediated by signaling through proteins ELMO, Dock180, and Rac1 [10]. Apart from its immune-system-related activities, BAI1 has a unique function as a PtdSer receptor to promote the fusion of healthy myoblasts via the same signaling module: proteins ELMO, Dock180, and Rac1 [55]. Myofibers of *Bai1^−/−^* mice are smaller than those from the wild type, thus highlighting the participation of BAI1 in myogenesis. Brain-specific angiogenesis inhibitor-1 could be an important target for further research into muscle growth or recovery after muscle injuries because BAI1 induces the fusion between healthy myoblasts through the interaction with its ligand: PtdSer exposed on apoptotic myoblasts [55].

Another BAI family member, BAI3, has also been identified as an essential transmembrane receptor interacting with ELMO-DOCK1 for vertebrate myoblast fusion [56]. That study indicates that the overexpression of BAI1 cannot reverse the myoblast fusion defects caused by the loss of BAI3, which means that BAI3 is functionally distinct from BAI1 [56]. Furthermore, BAI2 is unessential for myoblast fusion [56]. In the most recent study, it was also elucidated that BAI3 activity in myoblast fusion is spatiotemporally regulated by C1qL4 and stabillin 2, acting as a BAI3 ligand and its binding partner, respectively [57]. Both BAI1 and BAI3 can promote myoblast fusion via binding to ELMO-Dock1 during muscle development and repair (Figure 2C).

## 4. Brain-Specific Angiogenesis Inhibitors in Tumorigenesis

### 4.1. The Role of Brain-Specific Angiogenesis Inhibitor1 in Several Malignant Tumors

Many pieces of emerging evidence have shown that BAI1 is implicated in the progression of several malignant tumors [3,58,59,60]. As mentioned above, BAI1 was initially identified as a mediator of p53 activities in the brain, suggesting that BAI1 has an antiangiogenic function [1]. Hence, the function of BAI1 has been studied mostly in glioblastoma (a brain tumor), which is related to extensive vascular proliferation during its progression [61,62]. Brain-specific angiogenesis inhibitor-1 has been reported to be expressed in various tissues of a normal brain but has not been detected in the majority of glioblastomas and is entirely absent in glioma cell lines [3]. After radiation therapy, the clinical outcome of glioblastoma patients with BAI1 expression in the tumor is better than that of glioblastoma patients without this expression [63]. Furthermore, an in vivo study suggests that gene therapy using BAI1 may be a method for the treatment of human glioblastoma [64]. Tumor-implanted severe combined immunodeficiency (SCID) mice treated with an adenoviral vector encoding BAI1 (AdBAI1) show impairment of tumor growth and increased survival, suggesting that BAI1 could have antitumor effects due to its antiangiogenic properties [64].

The fragment cleaved by BAI autoproteolysis is related to tumor suppression [26,27,65]. Members of the BAI family contain a GPS motif, which includes a conserved serine/threonine residue [12]. Especially, Vstat120, a soluble 120 kDa fragment containing five TSRs, can be generated by cleavage at the GPS motif of BAI1 and can function as an antiangiogenic and antitumorigenic factor, suppressing tumor growth and vascular density in glioma-xenografted mice (Figure 2D) [65]. Vstat120 has a tumor-suppressive effect during the intracranial growth of malignant gliomas, which is accompanied by a decrease in the tumor vascular density both in vitro and in vivo via CD36: a cell surface receptor on endothelial cells [27]. Furthermore, an N-terminal 40 kDa fragment of BAI1, Vstat40, released by the cancer-associated protease matrix metalloproteinase 14 (MMP14), has been reported to suppress tumor angiogenesis and to delay tumor growth in a mouse model of a human tumor xenograft (Figure 2D) [26].

Recently, BAI1 was demonstrated to suppress medulloblastoma (MB) tumorigenesis by stabilizing p53 in a mouse MB model [66]. They reported that BAI1 is involved in the stabilization of p53 through blocking Mdm2-mediated p53 degradation, and the reactivation of BAI1 by an MBD2 pathway inhibitor protects p53 and inhibits MB growth in a mouse model [66]. This study suggests that the BAI1-p53 signaling axis may be effective against brain tumorigenesis and could be a therapeutic target in MB [66].

In addition to brain tumors, *BAI1* gene expression in pulmonary adenocarcinomas has been investigated to evaluate the relation between the expression level of BAI1 and vascular density. Vascular density has been found to be lower in BAI1-positive adenocarcinomas compared to BAI1-negative ones [59]. Similarly, BAI1 expression is significantly lower in colorectal cancers and inversely correlates with the vascularity of colorectal tumors [58,60]. In vivo tumor growth in renal carcinoma is reduced in mice inoculated with Renca/BAI1 (mouse renal cell carcinoma cells (Renca) transfected with BAI1) compared to the Renca wild type, suggesting that exogenous BAI1 can suppress tumor growth via the inhibition of angiogenesis [67]. A recent study also suggests that BAI1 expression is significantly reduced in breast cancer, and low BAI expression is associated with decreased survival [68]. Furthermore, oncolytic virus 34.5ENVE delivering Vstat120 enhances the survival of mice with intracranial breast cancer, suggesting that BAI1 is a putative therapeutic target in patients with breast cancer brain metastases [69].

### 4.2. The Roles of Brain-Specific Angiogenesis Inhibitor2 and Brain-Specific Angiogenesis Inhibitor3 in Tumors

Although the relevance of BAI2 and BAI3 to cancer has been less studied than that of BAI1, BAI2 has been found to be mutated in breast, lung, and ovarian cancers along with BAI1, and a mutation in BAI3 has been found in lung cancers through the systemic characterization of somatic mutations in the cancer genome [68]. The involvement of these somatic mutations in tumor biology remains to be elucidated. The relation of BAI3 with glioblastoma has not been reported so far. One study, however, has shown that the expression of BAI3 is decreased in an in vivo model of focal cerebral ischemia and in malignant glioma compared to the norm [15]. In recent years, a novel association between BAI3 and small cell lung carcinoma (SCLC) was uncovered. The *BAI3* gene is preferentially expressed in SCLC and has been identified as an immunohistochemical marker that distinguishes SCLC from large cell neuroendocrine lung carcinomas [70]. Moreover, a nuclear staining pattern of BAI3 can be considered evidence of an SCLC phenotype, although the mechanism of aberrant nuclear expression remains poorly understood.

## 5. Brain-Specific Angiogenesis Inhibitors in Neurological Diseases

### 5.1. Brain-Specific Angiogenesis Inhibitor1

A study on BAI1 related to neurovascular injury by ischemia induction suggests that *BAI1* expression decreases on the ischemic side by 24 h postischemia induction, indicating that BAI1 is involved in the suppression of angiogenesis and neuronal differentiation for revascularization of the damaged brain tissue [7].

Recently, BAI1 has been implicated in synaptogenesis and synaptic plasticity (Figure 2E) [11,71,72]. Small GTPase Rac1 is known to modulate the development and plasticity of synapses and neuronal spines; these phenomena are promoted by polarity protein Par3, which spatially controls Rac1 activation [73,74]. BAI1 interacts with the Par3-Tiam1 complex (Tiam1 is a Rac1-guanine nucleotide exchange factor), and this interaction modulates synaptogenesis by regulating the localization of the Par3-Tiam1 polarity complex at synaptic sites and thus induces polarized Rac1 activation and cytoskeleton remodeling [11]. Brain-specific angiogenesis inhibitor-1, highly enriched in a postsynaptic density fraction, is a synaptic receptor and its C terminus can interact with several PDZ domain–containing synaptic proteins (MAGI-1, MAGI-3, and PSD-95), which can regulate various signaling cascades downstream of BAI1 [32]. In particular, PSD-95, a synaptic scaffolding protein, has been reported to be degraded for synapse elimination, which is related to Fragile X syndrome manifesting an abnormality of the dendritic spine number [75,76]. Additionally, PSD-95 has been found to be downregulated by β-amyloid accumulation in amyloid precursor protein (APP)-mutant neurons, which is characteristic of Alzheimer’s disease, suggesting that the dysregulation of PSD-95 could be involved in Alzheimer’s disease pathogenesis [77,78]. Another interesting BAI1-binding protein is insulin receptor substrate 53 (IRSp53), also known as BAI1-associated protein 2 (BAIAP2), which binds to the proline-rich region in the BAI1 C terminus and is highly expressed in the postsynaptic density of excitatory synapses [79]. This protein has been reported to be closely associated with autism susceptibility and adulthood attention deficit hyperactivity disorder [79]. Therefore, BAI1 could be a therapeutic target because of its significance linked to its binding partners, which are associated with neurological disorders.

### 5.2. Brain-Specific Angiogenesis Inhibitor2

Brain-specific angiogenesis inhibitor-2, like BAI1, is linked to angiogenesis in the injured brain after ischemic stroke. In a focal ischemia model based on occlusion of the cerebral artery, *BAI2* expression decreases in the ischemic cerebral cortex after ischemia induction, and the expression level of BAI2 inversely correlates with that of a vascular endothelial growth factor (VEGF), which is a key mediator of angiogenesis [13]. Further study has also shown that BAI2 regulates *VEGF* transcription through its association with GABP (GA-binding protein), which works as a transcriptional repressor during cerebral ischemia, as well as under normal conditions [33].

Participation of BAI2 in psychiatric problems has been uncovered in BAI2-deficient mice, which manifested an increase of motor activity in the social defeat test and a decrease of immobility time in the tail suspension test compared to wild-type mice [80]. These data suggest that BAI2 could play an important role in mood disorders, although the molecular mechanisms relevant to the antidepressant-like effects in BAI2-deficient mice have yet to be elucidated.

Recently, BAI2 was implicated in a neurological disorder, specifically, in progressive spastic paraparesis [81]. C-terminal mutation in BAI2 was demonstrated to increase the signaling activity of this receptor in vitro. This study suggests that an increase in BAI2 activity may lead to neuromuscular diseases [81].

### 5.3. Brain-Specific Angiogenesis Inhibitor3

A decrease in *BAI3* expression has been detected in a focal ischemia model, as is the case for BAI1 and -2. The reduction of the BAI3 level occurs from 30 min to 8 h after the induction of hypoxia, suggesting that BAI3 could be regulated in the early phase of ischemia-induced brain injury [15].

In recent years, BAI3’s function in the excitatory synapses of the brain has been studied extensively. Initially, biochemical studies revealed that BAI3 binds to the C1ql protein that is expressed and secreted in the brain, and this interaction is important for the regulation of synapse formation and maintenance [31]. Further research has revealed that the interaction between C1ql1 from climbing fibers and BAI3 on postsynaptic Purkinje cells is crucial for determining single-winner climbing fibers during the development and elimination of weak climbing fibers in adult mice [18]. This finding is consistent with the previous reports that both C1ql1 and BAI3 proteins are highly expressed during synaptogenesis, and their interaction controls the synapse connectivity of cerebellar Purkinje cells [17]. Furthermore, it has been demonstrated that the expression of BAI3 is enhanced in Purkinje cells, and C1ql1, as a ligand of BAI3, regulates the spinogenesis of Purkinje cells in a BAI3-dependent manner (Figure 2E) [17]. Recently, BAI3, as a receptor of Clql3, was reported to be involved in the inhibition of insulin secretion from pancreatic β-cells [82]. This result suggests that BAI3 signaling might be linked to type 2 diabetes [82].

Brain-specific angiogenesis inhibitor-3 participation in mental disorders has been investigated by the genetic analysis of symptom-based phenotypes. Two-single nucleotide polymorphisms (SNPs) within *BAI3* are significantly associated with the disorganization symptom of schizophrenia according to genetic linkage studies [83]. Copy number changes in chromosomal region 6q12 (encoding BAI3) have been found in familial schizophrenia [84]. Additionally, BAI3 expression is changed by treatment with lithium carbonate, an effective drug for bipolar disorder [85]. Brain-specific angiogenesis inhibitor-*3* has also been reported as one of the clock modulator genes that are closely linked to the bipolar disorder spectrum and lithium response [86], suggesting that BAI3 could be a target for the treatment of psychiatric and neurological disorders.

## 6. Conclusions

It has been shown that BAI1, a member of the adhesion GPCR family, functions as an engulfment receptor in professional and nonprofessional phagocytes, inhibits angiogenesis and tumorigenesis, and regulates synaptogenesis and spinogenesis. Therefore, it is not surprising that BAI proteins have been implicated in a variety of human diseases. This review provides an overview of the BAI subfamily of adhesion GPCRs, with a focus on uncovered critical roles of the BAI subfamily members that are involved in human diseases. The converging evidence that BAIs participate in human diseases suggests that these receptors could be new therapeutic targets in a variety of diseases, although our understanding of the complex roles of BAI family proteins in various pathological and physiological conditions is insufficient. To determine the therapeutic utility of the BAI family proteins, a complete understanding of the pharmacological properties of these receptors is required through structure-based studies of the BAI family. Class B GPCRs, which BAI family receptors belong to, are generally considered drug targets in human diseases. The first step toward the targeting of the BAI family receptors is the identification of all BAI ligands that bind to the large N-terminal region in these receptors, which can mediate cell-to-cell interaction or communication. High-throughput screening to find additional ligands for BAI receptors may be a feasible method for identifying small-molecule compounds that function as agonists or antagonists of BAIs as receptors in various pathological and physiological conditions. The second step includes complete elucidation of the downstream signaling pathways of BAIs as receptors. Preferential activation of the downstream pathways of BAIs by each potential BAI ligand, in concert with intracellular interacting partners for BAIs in a broad spectrum of pathological and physiological conditions, needs to be clarified. Lastly, the regulatory role and dynamics of BAI family proteins according to cell types, intracellular locations, developmental stages, and physiological states should be taken into account for the future therapeutic uses of BAIs, beyond all the currently available knowledge on BAIs.
